# Influence of In-Situ Stress on Cut Blasting of One-Step Raise Excavation Using Numerical Analysis Based on a Modified Holmquist-Johnson-Cook Model

**DOI:** 10.3390/ma16093415

**Published:** 2023-04-27

**Authors:** Kai Liu, Qiyue Li, Chengqing Wu, Xibing Li, Wei Zhu

**Affiliations:** 1 School of Resources and Safety Engineering, Central South University, Changsha 410083, China; kailiu30@csu.edu.cn (K.L.);; 2 School of Civil and Environmental Engineering, University of Technology Sydney, Sydney, NSW 2007, Australia

**Keywords:** cut blasting, damage model, in-situ stress, numerical simulation, field test

## Abstract

Due to different tensile and compressive properties of rock material, the corresponding tensile and compressive damage evolution show major differences. To investigate the tensile and compressive damage evolution in deep cut blasting with different in-situ stresses, an improved Holmquist-Johnson-Cook (HJC) material model considers the tensile and compressive damage separately is developed. The improved HJC model is implemented into LS-DYNA via a user-defined subroutine in this study. Then, a numerical model with different in-situ stresses loading schemes is modelled. Numerical simulation results show that in-situ stress can inhibit the development of tensile damage evolution, while promote the development of compressive damage evolution. The overall damage zone presents a decreasing trend with the increase of in-situ stress, because the tensile damage is more sensitive than the compressive damage for rock material. In addition, the maximum principal stress can determine the development of the direction of damage. Further, for a field test of blind cut raise in deep, the actual in-situ stress values are loaded on the numerical model. Then, in order to overcome the difficulties caused by in-situ stress, the cut blasting design is optimized by reducing hole spacing. Subsequently, the optimized cut parameters are applied in the blind cut raise. However, the one-step raise excavation method is adjusted to two steps to ensure success due to a serious borehole deviation between drilling and design drawing. After these steps, the formation of the blind cut raise with 8.7 m depth is met the requirements of design.

## 1. Introduction

Raises such as ventilating raise, cut raise and service raise is widely used in underground space engineering [1,2,3]. However, conventional raise excavation methods, which need workers to gain access to the raise heading face and drill 3~4 m per excavation cycle, are insecure and inefficient. Compared with the conventional raise excavation methods, the one-step raise excavation technique has huge advantages in security, cost and efficiency [4]. Thus, it has been gradually applied in the field of underground engineering. The one-step raise excavation technique can be divided into two styles based on charging structure: spherical cartridge blasting mode (SCBM) and burn cuts blasting mode (BCBM) [5]. Compared to SCBM, the BCBM is more flexible in underground engineering, especially in deep mines with narrow space. Meanwhile, BCBM has more advantages in excavation of small section raise. The BCBM needs a cut cavity, which is formed by initiation of a series of cut holes in sequence, to serve as the free surface and swelling space for the boreholes [6]. Thus, it is crucial to investigate the damage evolution process of cut-blasting. When cut blasting is performed in a deep mine, damage induced by blasting load is affected by not only cut parameters but also high in-situ stress which will cause difficulties to cut blasting [7,8,9]. As a result, the application of one-step raise excavation is limited in deep mines. Therefore, it is necessary to study damage evolution and failure mechanisms of cut blasting under high in-situ stress.

In recent years, many studies on damage evolution of rock mass under blasting loading have been conducted [10,11]. Liu and Katsabanis [12] described a constitutive model for predicting rock damage and fragment size distribution based on continuum mechanics and statistical fracture mechanics. Zhang et al. [13] proposed an anisotropic continuum damage model to study rock damage induced by stress wave. In addition, some damage models are used in the numerical simulation of cut blasting. Yang et al. [14] implemented a statistical damage evolution law into LS-DYNA to simulate rock damage evolution of deep tunnel excavation. Xie et al. [15] developed a tension and compression-shear damage model to investigate the damage mechanics of cut blasting under high in-situ stress. However, little work on one-step raise excavation has been done except that covering tunneling excavation. The cut model, excavation depth, section and direction are different between raises and tunnels, which will result in differences in the damage evolution of rock mass during cut blasting [4].

It is well-known that rock material exhibits different damage evolution mechanisms under tension and compression loading. However, some damage constitutive models cannot describe well the differences between tensile damage and compressive damage [16,17,18]. For example, the Holmquist-Johnson-Cook (HJC) model can express compression damage behavior via its damage model, which defines damage accumulates from equivalent plastic strain and plastic volumetric strain, but the tension damage behavior is not taken into account in the HJC model [19]. Due to the tensile strength of rock mass which is much less than its compressive strength, the tensile damage is the main cause for rock failure. Therefore, many damage-constitutive models have been proposed and implemented to describe both the compressive and tensile damage evolution mechanisms of rock mass. Hao et al. [20] present a numerical model, in which both the tensile and compressive damage are involved, to simulate the damage zone around the charge hole. Li and Shi [21] established a tensile and compressive damage model based on the extended Drucker-Prager strength criterion and the Johnson–Cook material model. Considering that the compression behavior of materials was described in detail and a few parameters needed to be determined, the original HJC model is modified in this study.

The objective of this study is to investigate the damage evolution mechanism of cut blasting in the BCBM under different in-situ stresses. Firstly, a modified HJC damage model which considers tension and compression damage behaviors is presented and implemented into LS-DYNA software (R 11.0, Sydney, Australia) via user subroutines. After this, for deep raise excavated by the BCBM, a simplified cut blasting numerical model is built to analyze the behaviors of rock damage under different in-situ stress load schemes. Further, the actual in-situ stresses in −665 m sublevel of a gold mine are applied to the numerical model to obtain the optimized cut blasting parameters. Finally, the optimization parameters are applied to the field test of BCBM.

## 2. Engineering Background

### 2.1. General

**Figure 1:**
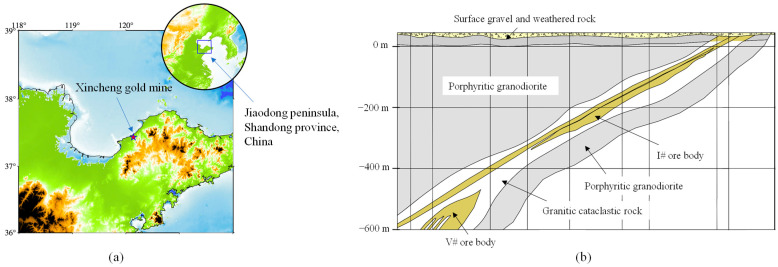
Basic information of Xincheng gold mine: (**a**) the location of Xincheng gold mine; (**b**) the engineering geology profile of Xincheng gold mine.

Xincheng gold mine is located in Laizhou city, Shandong province, China. The mine area is about 35 km southwest of Laizhou city (37°25′ N, 120°08′ E). Via the G206 national road which runs through the mine area, the mine is connected with the Weifang railway station about 135 km to the southwest, as shown in Figure 1a. Xincheng gold mine is a medium mechanized underground mine with a production capacity of over 3600 t/day. The ore deposit is mainly composed of I# and V# ore bodies in Xincheng gold mine. The I# ore body, which is located at a depth from −20 m to −630 m, has been exhausted. Therefore, the burden of the main ore output is carried by the V# ore body which is located at a depth from −430 m to −900 m, as shown in Figure 1b. In order to improve the production capacity of Xincheng gold mine, a new mining method should be determined for the V# ore body. Because the cut and fill stoping method has been used in the thin ore body I#, it is no longer suitable for the large and thick ore body V#. Sublevel fill stoping, which is a mass mining method and can meet the production requirement [22,23], will be adopted for the V# ore body. For sublevel fill stoping, a vertical cut raise should be excavated as the free surface for production holes blasting. Therefore, the BCBM is used in construction of the cut raise to improve excavation efficiency and ensure operational safety.

### 2.2. Design of Cut Blasting for One-Step Raise Excavation

**Figure 2:**
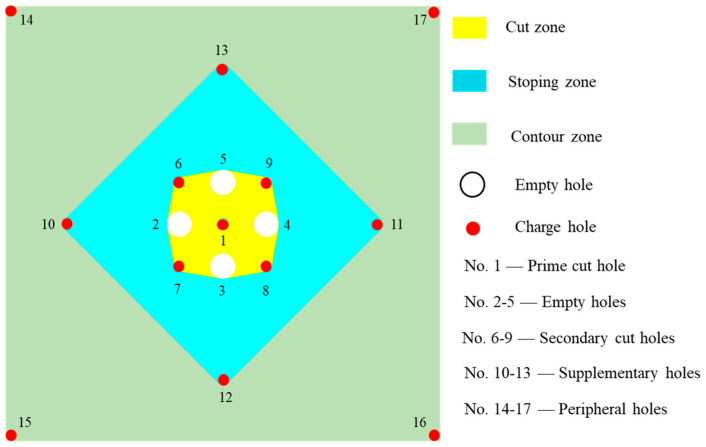
Layout of boreholes for burn cuts with four empty holes.

According to the surrounding rock mass stability and sublevel height, a blind cut raise with a height of 9 m and a cross section of 2.5 × 2.5 m^2^ is designed. The BCBM is used to excavate the deep cut raise in this study. For the application of the BCBM, cut model and parameters should be determined firstly, especially for the prime cut hole which detonates firstly and uses empty holes as the free surface and swelling space in the process of cut blasting. However, the constriction of burden rock in cut-blasting is much higher than that in bench blasting. Moreover, high in-situ stress will cause more difficulties for cut blasting in deep mines. According to the research of Xie et al. [15], the empty hole not only plays the role of free surface but can also transfer the blasting energy to the free surface. Consequently, more empty holes applied in the cut model means more blasting energy will be used to break rock mass. Thus, a burn-cut-with-four-empty-holes model is adopted for the BCBM in a deep mine, as shown in Figure 2, which includes one prime cut hole (No. 1), four empty holes (No. 2, 3, 4, and 5) and four secondary cut holes (No. 6, 7, 8, and 9) in the cut zone, and four supplementary holes (No. 10, 11, 12, and 13) and four peripheral holes (No. 14, 15, 16, and 17) in the stoping and contour zones. It can be seen that as the empty holes are arranged around the prime cut hole, more blasting energy will be shifted to the direction of empty hole and spent in the creation of the cut cavity after the prime cut hole detonation. The sizes of boreholes are determined based on the existing drilling equipment in Xincheng gold mine. The diameters of charge holes and empty holes are 70 mm, 130 mm respectively. According to the requirement of swelling coefficient *k*, the hole spacing *L* between the prime cut hole and empty holes is set as 250 mm to ensure *k* > 1.45 [24].

## 3. Material Model for Numerical

### 3.1. Blasting Damage Model for Rock Material

As we all know, the explosive energy can convert into two main forms: stress waves, and detonation gas after it is initiated. The rock damage induced by blasting also results from the two forms. Nevertheless, it is difficult to quantify the effects of the two forms and describe the two damage mechanisms in a single damage model. Thus, the effects of stress waves and detonation gas on rock damage are generally independently modeled in most studies [25,26]. The damage evolution models produced by detonation gas cannot give a reasonable prediction in numerical simulation and have not been further pursued [27]. Meanwhile, the damage models only consider the effect of stress waves can predict the rock respond to blasting load. Thus, the blasting damage model only induced by stress waves is modeled in this study.

#### 3.1.1. Damage Evolution Model

For brittle material, such as rock or concrete, its compressive strength is much greater than its tensile strength. In the process of explosion stress wave propagation, the compressive component derived by shock wave produces compressive-shear damage in the vicinity of charging hole. After this, a tensile damage zone will appear outside the compressive-shear damage zone with the propagation of stress wave. Due to different mechanical properties under tensile and compression loads, the rock damage evolution model can be divided into two parts: tensile damage function and compressive damage function. When the rock material is in a tensile state, its damage evolution should be expressed by the tensile damage function. Conversely, its damage evolution can be expressed by the compressive damage function.

In major reported studies on tensile fracture of the brittle material [28,29], the stress-strain curve under uniaxial tension is similar to the exponential form in the tensile softening stage. Therefore, referring to the study of Weerheijm and Doormaal [29], an exponential softening expression in which the tensile damage *D*_t_ is accumulated with plastic strain is adopted, as following,

(1) $$D_{t}=\left[1+{\left(c_{1}\frac{{\bar{\varepsilon }}_{P}}{\varepsilon_{\mathrm{frac}}}\right)}^{3}\right]\exp\left(-c_{2}\frac{{\bar{\varepsilon }}_{P}}{\varepsilon_{\mathrm{frac}}}\right)-\frac{{\bar{\varepsilon }}_{P}}{\varepsilon_{\mathrm{frac}}}\left(1+c_{1}^{3}\right)\exp\left(-c_{2}\right)$$

where, ${\bar{\varepsilon }}_{P}=\sum \Delta \varepsilon_{p}$ is the effective plastic strain, $\varepsilon_{\mathrm{frac}}$ is the fracture strain which is depended on the element size in the numerical model. The constants *c*_1_ = 3 and *c*_2_ = 6.93. $\varepsilon_{\mathrm{frac}}$ is the fracture strain, and its value depends on the size of the element in the numerical model. It can be calculated by

(2) $$\varepsilon_{\mathrm{frac}}=\frac{5.136G_{f}}{h_{c}T}$$

where *G*_f_ is the fracture energy and is taken as 80 Nm/m^2^ from Kong et al. [19]. *h*_c_ is the characteristic length of the element, which may be approximated by the cube root of the volume of the element in a 3D analysis. *T* is the tensile strength of material.

For compressive damage evolution, not only the plastic strain which is caused by plastic shear deformation but also the plastic volume strain which is induced by plastic crushing of the pores in the rock mass should be considered in compression damage accumulation. According to the definition in the HJC model, the compressive damage *D*_c_ is proposed as follows,

(3) $$D_{c}=\sum \frac{\Delta \varepsilon_{p}+\Delta \mu_{p}}{\varepsilon_{p}^{f}+\mu_{p}^{f}}$$

where, $\Delta \varepsilon_{p}$, $\Delta \mu_{p}$ are the effective plastic strain increment and plastic volumetric strain, respectively, during a cycle of integration. $\varepsilon_{p}^{f}+\mu_{p}^{f}$ is the total plastic strain under a constant pressure until fracture, which is expressed as,

(4) $$\varepsilon_{p}^{f}+\mu_{p}^{f}=D_{1}{\left(p^{*}+T^{*}\right)}^{D_{2}}\geq EF_{\min}$$

where, *D*_1_ and *D*_2_ are the damage constants. $p^{*}=\frac{p}{f_{c}}$, $T^{*}=\frac{T}{f_{c}}$ denote the normalized pressure and the normalized hydrostatic tension respectively, and *p* is the actual pressure, *T* is the maximum hydrostatic tension, *f*_c_ is the quasi-static uniaxial compressive strength. $EF_{\min}$ is used to suppress fracture from low tensile stress waves. 

In order to show which kind of damage predominates in the failure of rock mass, the maximum function is applied. Thus, the final damage variable can be expressed as,

*D* = Max (*D*_c_, *D*_t_) (5)

#### 3.1.2. Yield Strength Model

Just like many previous studies, the shape of the yield surface of the brittle material in the deviatoric plane exhibits a triangular shape at low pressure and transforms to a circular shape at high pressure. However, many material models such as the classic Drucker–Prager model [21] and HJC model [19] have assumed that the shape of yield surface remains a circle the whole time in the deviatoric plane. That means there are not differences between the tension meridian and the compression meridian, which result in the consequence that the capacity of rock subjected to tension loading will be overestimated in the above models. Therefore, the Lode-angle function [30] depending on Lode-angle (*θ*) and *e* should be introduced to the yield strength surface, which is expressed as,

(6) $$R\left(\theta ,e\right)=\frac{2\left(1-e^{2}\right)\cos\theta +\left(2e-1\right){\left[4\left(1-e^{2}\right)\cos^{2}\theta +5e^{2}-4e\right]}^{\frac{1}{2}}}{4\left(1-e^{2}\right)\cos^{2}\theta +{\left(2e-1\right)}^{2}}$$

where, the Lode-angle *θ* is determined by,

(7) $$\theta =\frac{1}{3}\cos^{-1}\left(\frac{27J_{3}}{2\sigma_{\mathrm{eq}}^{3}}\right)$$

with *J*_3_ being the third invariant of the deviatoric part of the stress tensor, $\sigma_{\mathrm{eq}}$ being the equivalent stress. *e* is the ratio of the tensile meridian to the compressive meridian, which can describe the transition of deviatoric plane shape from triangle to circle. As discussed by Polanco-Loria et al. [30], the *e* which is sensitive to the biaxial-compression strength is simply defined by a linear interpolation of the discrete points, as follows

(8) $$e=\left\{\begin{array}{l} 0.65\;\;\;\;\;\;\;\;p^{*}<0\\ 0.65+\left(1-0.65\right)\frac{p^{*}}{p_{\mathrm{ref}}^{*}}\;0\leq p^{*}\leq p_{\mathrm{ref}}^{*}\\ 1.0\;\;\;\;\;\;\;\;\;\;p^{*}>p_{\mathrm{ref}}^{*}\; \end{array}\right.$$

where, $p_{\mathrm{ref}}^{*}$ is the reference normalized pressure and $p_{\mathrm{ref}}^{*}=10$ is adopted in this study. 

#### 3.1.3. Strain Rate Effect Model

The strain rate effect is used to describe the phenomenon that material strength increases with strain rate under dynamic loading. In order to describe the strain rate effect in the constitutive model, a simplified approach in which the static yield surface is multiplied by the dynamic increase factor (*DIF*) as the dynamic yield surface is implemented. However, the *DIF* should be defined separately for the tension and compression loading, since dynamic experimental results show that the *DIF* for tension is much higher than that for compression. Many tension-compression strain rate effect expressions have been put forward recently [31]. However, most of the expressions are of a exponential relationship, which will lead to an overestimate of *DIF* with the strain rate increases to the range of 10^4^ s^−1^~10^6^ s^−1^. Gebbeken and Greulich [32] suggested a hyperbolic function to describe the relationship between strain rate and *DIF*, which sets a cut-off of *DIF* in high strain rate region and shows a good agreement with the experiment data of brittle material.

(9) $$DIF_{t}=\{[\tan h((\mathrm{lg}(\dot{\varepsilon }/{\dot{\varepsilon }}_{0})-W_{x})S)][\frac{F_{m}}{W_{y}}-1]+1\}W_{y}$$

(10) $$DIF_{c}=\left(DIF_{t}-1\right)\left(T/f_{c}\right)+1$$

in which, *DIF*_t_ and *DIF*_c_ are the dynamic increase factor for tension and compression, respectively. $W_{x}$, S, $F_{m}$, $W_{y}$ are the fitting constants, the values of *W_x_* = 1.6, *S* = 0.8, *F_m_* = 10, *W_y_* = 5.5 are determined referred the research of Tedesco et al. [33], ${\dot{\varepsilon }}_{0}=1s^{-1}$ is the reference strain rate. In this study, the above hyperbolic function for strain rate effect is adopt. Thus, according to the form of continuous yield surface function proposed by Polanco-Loria et al. [30], it can be written as follows,

(11) $$\sigma^{*}=\left\{\begin{array}{c} B{\left[T^{*}(1-D)+p^{*}\right]}^{N}R\left(\theta ,e\right)DIF,\;P^{*}\geq -T^{*}(1-D)\;\\ \;\;\;\;\;\;0\;\;\;\;\;\;\;\;,\;P^{*}<-T^{*}(1-D) \end{array}\right.$$

where, *B* is the pressure hardening coefficient, *N* is the pressure hardening exponent, and the other parameters are the same as above. In addition, a typical three-stage equation of state for brittle material is used to describe the relationship between pressure and volume strain [19,21]. The three-stage equation of state contains bulk modulus *K*, pressure parameter *K*_1_, *K*_2_ and *K*_3_, hydrostatic pressure $p_{c}$ and volume strain $\mu_{c}$ at the elastic limit, $p_{l}$ and $\mu_{l}$ at the crushing region. A detailed description of this modified HJC model and its validation can be found in Ref. [34], which is a previous study of the authors.

### 3.2. JWL EOS for Explosive

The borehole pressure profiles can be approximated using two main methods: pressure-decay functions and Equation-of-State (EOS), while the EOS can describe the process of rock-explosive interaction and is convenient for application in LS-DYNA. Therefore, the JWL EOS is used to simulate detonation products of high explosives in the present study. The JWL EOS defines the pressure as:

(12) $$P_{J}=A_{J}(1-\frac{\omega }{R_{1}V})e^{-R_{1}V}+B_{J}(1-\frac{\omega }{R_{2}V})e_{1}{}^{-R_{2}V}+\frac{\omega E}{V}$$

where $P_{J}$ is the pressure of the detonation products, V is the relative volume of detonation products, E is the special internal energy with an initial value of $E_{0}$, $A_{J}$, $B_{J}$, $R_{1}$, $R_{2}$ and ω are material constants. 

### 3.3. Air Material Model

In addition, the radial decoupling charge is usually applied on cut blasting to control the blasting damage zone in practice. In this study, the radial air-decoupling charge technique is implemented. As for air, material type 9 of LS-DYNA (*MAT_NULL) is used to calculate the pressure from a specified EOS, which is expressed as:

(13) $$P_{A}=C_{0}+C_{1}\delta +C_{2}\delta^{2}+C_{3}\delta^{3}+(C_{4}+C_{5}\delta +C_{6}\delta^{2})e_{2}$$

where *P*_A_ is the pressure, $e_{2}$ is the internal energy per volume, *δ* is dynamic viscosity coefficient, $C_{0}$, $C_{1}$, $C_{2}$, $C_{3}$, $C_{4}$, $C_{5}$ and $C_{6}$ are material constants. In addition, the air is modeled as an ideal gas by setting $C_{0}=C_{1}=C_{2}=C_{3}=C_{6}$ = 0 and $C_{4}=C_{5}=0.401$, and air mass density and initial internal energy are 1.255 kg/m^3^ and 0.25 J/cm^3^, respectively [35].

## 4. Numerical Simulation and Analysis

### 4.1. Research Methodology and Steps

**Figure 3:**
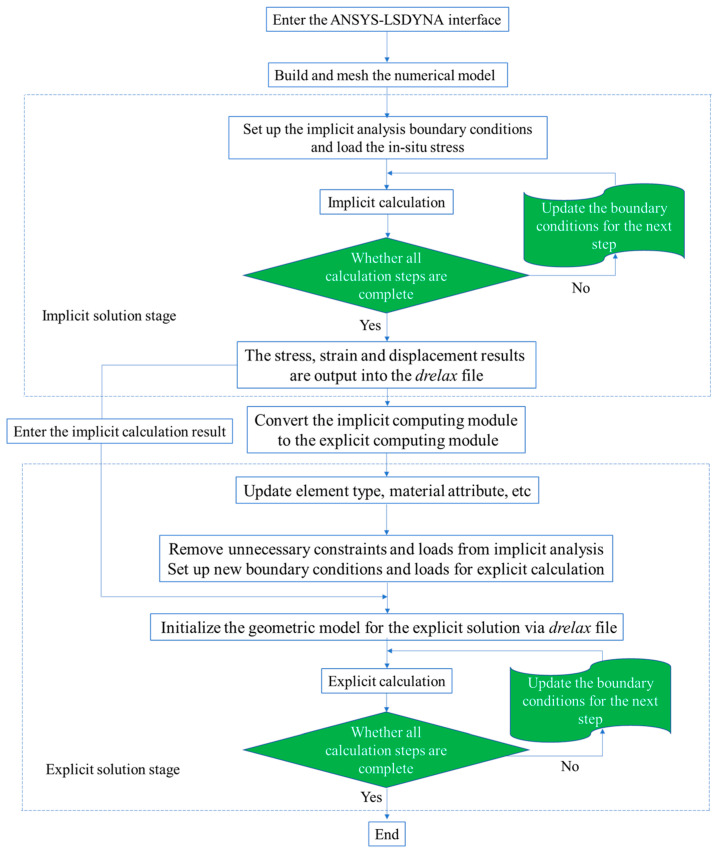
Flow chart of the implicit-explicit sequential solution.

The numerical software ANSYS-LSDYNA is used to simulate and reproduce the whole process of cut blasting under in-situ stress [36]. In order to load in-situ stress at the boundary of the numerical model, the implicit-explicit sequential solution method is involved. First, the implicit module is called to analyze the static in-situ stress state of the cut blasting model. Then the implicit calculation result contains strains, displacements and stresses is output as a *drelax* file which will be imported to the explicit module during the explicit calculation. In the explicit stage, the boundary conditions and element type should be updated. The unnecessary constrains are deleted from the implicit analysis and the new boundary conditions are set up for explicit solution. Then the numerical model is initialized for the explicit solution via the *drelax* file, as shown in Figure 3. The element type should be transformed from solid 185 in implicit to solid 164 in explicit. Besides, in the explicit analysis stage, there are basic materials such as rock, explosive to be modeled. In the process of high-speed explosion, the explosives can be treated as fluid and modeled with Euler mesh, while the rock material is modeled with Lagrangian mesh in the ALE algorithm. The interaction between explosive and rock is achieved by the keyword “*CONSTRAINED_LAGRANGE_IN_SOLID”.

### 4.2. Numerical Model and Material Paramaters

**Figure 4:**
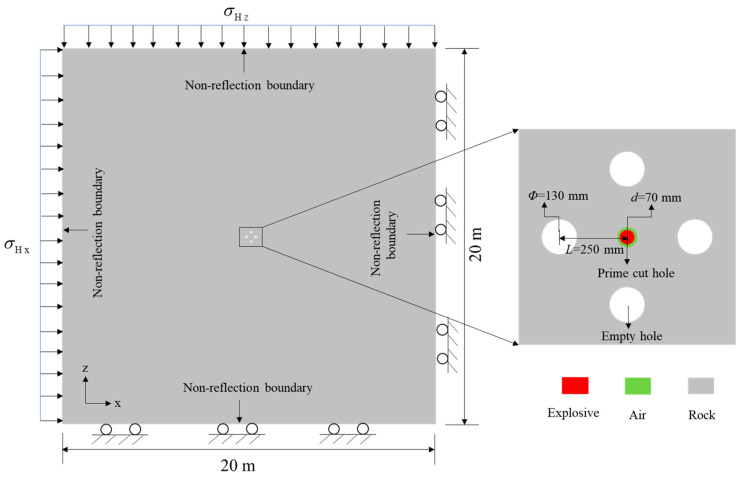
Geometric model and boundary conditions of the prime cut hole for the BCBM.

The damage evolution process of the prime cut hole is only taken into account in the numerical simulation. Because it is the most critical and difficult step in the cut blasting in which the empty holes serve as the only free surface for the prime cut hole [6]. Therefore, a prime cut numerical model with the dimension of 20 × 20 m^2^ (length × width) is modelled based on the cut blasting design, as shown in Figure 4. According to the influence of stress wave reflection and the crack zone of rock blasting [37,38], the numerical model size is set to 20 × 20 m^2^ (length × width). To simplify the problem, the numerical model is assumed as a plane strain problem, provided that the mechanical characteristics are the same in every cross section along the cut raise’s centerline. Thus, the numerical model can set as a three-dimensional single layer mesh model, which can improve the accuracy of the simulated results and reduce the computation time [39,40]. In addition, it can be seen that the parameters of hole diameters and hole spacing are consistent with the design. The radial air decoupling charge structure with 10 mm air layer is also used for the prime cut hole. In the implicit solution stage, initial horizontal in-situ stresses *σ*_Hx_ and *σ*_Hz_ are applied to the x and z direction faces of the numerical model, and the other two opposite surfaces are fixed boundaries. In the explicit solution stage, the unnecessary loads and constrains are deleted, and the non-reflection boundary conditions are set up in the four outerrounded faces to reduce the influence of stress wave reflection.

For the rock material, the basic parameters, such as density, uniaxial compressive strength, uniaxial tensile strength and shear modulus, are obtained by rock mechanics experiments. The other parameters such as yield surface strength parameters, equation-of-state parameters and damage parameters can be determined by referring to the author’s previous research [34]. The all-parameter values for the modified HJC model that need to be inputted into the LS-DYNA are presented in Table 1. Besides this, the JWL EOS parameters of explosive are listed in Table 2.

### 4.3. Damage Evolution under Different In-Situ Stresses

In order to investigate the effect of in-situ stress on rock damage evolution mechanisms of cut blasting during deep raise excavation, the corresponding loading schemes of in-situ stress are established. Since the current mining depth of Xincheng gold mine is 1200 m, the corresponding horizontal maximum in-situ stress is about 60 MPa, so the numerical simulation research scheme sets the values of in-situ stress within the range of 0–60 MPa, as shown in Table 3. Schemes 1–4 are used to study the effect of the hydrostatic pressure, while Schemes 5–8 are used to study the effect of the anisotropic in-situ stress. 

**Figure 5:**
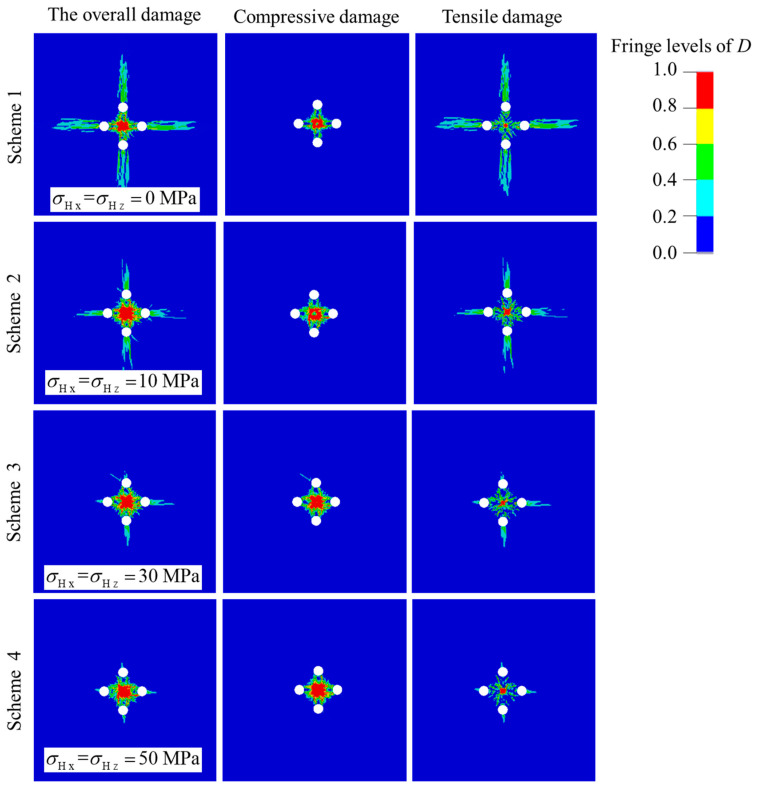
Damage distribution of prime cut blasting under the action of hydrostatic pressure.

The numerical simulation results of Schemes 1–4 are presented in Figure 5. It can be seen that with the increase of in-situ stress, the area of the overall damage tends to decrease gradually. That indicates that in-situ stress can restrain the development of rock damage evolution in cut blasting. Further, the tensile damage and compressive damage are separately analyzed. It is obvious that the area of the tensile damage drops, especially in the stress waves superposition zone behind the empty holes, with the increase of in-situ stress, while that of the compressive damage appears to rise. It is demonstrated that in-situ stress can promote the development of compressive damage, while inhibit the development of tensile damage. The main reason is that the tensile component of stress wave is suppressed by in-situ stress, which causes the tensile damage reduced significantly. When the stress wave propagates to the superposition zone behind the empty holes, the attenuation is more serious. Thus, the tensile damage area of the region decreases most obviously. In addition, when in-situ stress is below 30 MPa, the tensile damage zone drops sharply with the increase of in-situ stress. Nevertheless, when in-situ stress is higher than 30 MPa, the gradient of tensile damage zone drops is lower than the previous. The probably reason for this phenomenon is that when in-situ stress is higher than 30 MPa, the tensile component of stress wave in the superposition zone is reduced by in-situ stress and is lower than the dynamic tensile strength of rock. Therefore, in the mining industry, the linear charge density in deep mines is greater than that in shallow mines, to rise the peak value of shock wave and overcome the difficulties from in-situ stress.

**Figure 6:**
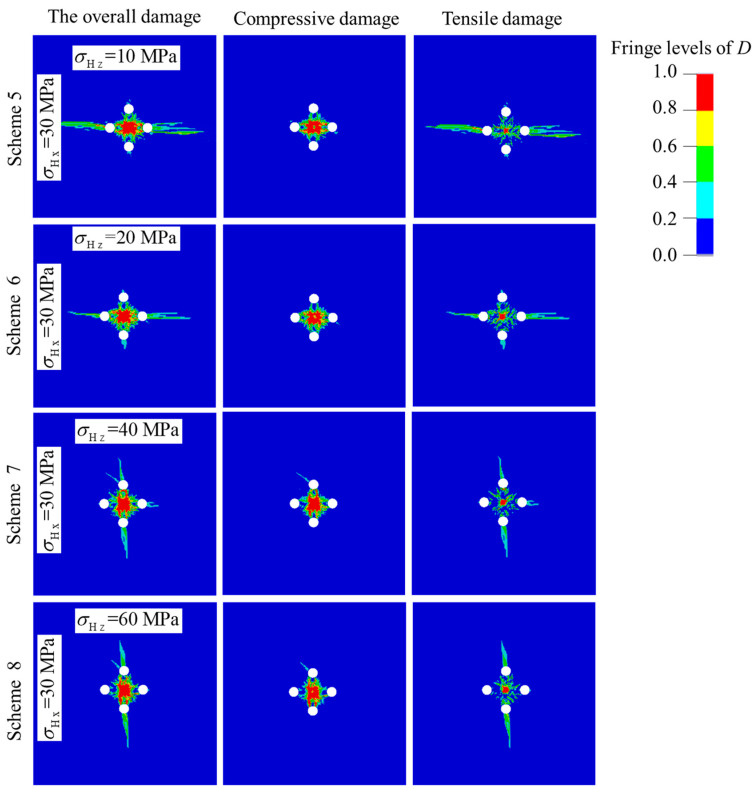
Damage distribution of prime cut blasting under the action of anisotropic in-situ stress.

Figure 6 shows the rock damage evolution of cut blasting under the action of anisotropic in-situ stresses. The results demonstrate that both the compressive damage and tensile damage are aligned with the direction of the maximum principal stress, and the phenomenon becomes more and more obvious with the increase of difference between the two principal stresses. The compressive damage shows a slight increase with the increase of in-situ stress, while the tensile damage drops sharply. The main reason for this is that the tensile stress component perpendicular to the maximum principal stress is greatly suppressed, while the tensile stress component perpendicular to the minor principal stress is slightly suppressed. In contrast, the compressive stress component is promoted by in-situ stress.

Therefore, for the cut blasting in deep mines, in order to overcome the suppressive effect of in-situ stress on tensile damage evolution, the simple and effective method is to reduce the hole spacing between the prime cut hole and empty holes [7], that is, to reduce the burden. According to the numerical simulation results, the burden can be reduced slightly in the direction of the maximum principal stress, while it needs to be reduced to a greater extent in the direction of the minor principal stress. Similarly, the promoting effect of in-situ stress on compressive damage evolution can be applied. If in-situ stress is high enough, the compressive component will be greatly promoted and the burden can be increased appropriately. For a project to determine the blasting parameters, such factors needs to be considered as the magnitude of in-situ stress and rock mechanical properties, and then using these to optimize the parameters through numerical simulation.

### 4.4. Case Study of Cut Parameters Optimization for Deep Raise

The experimental stope is located in −665 m sublevel, V# ore body, Xincheng gold mine. According to the literature [41], a study of in-situ stress distribution in Xincheng gold mine was conducted by Cai et al. [41]. It was found that in-situ stress field is dominated by horizontal tectonic stress rather than gravity stress in Xincheng gold mine. The measured results show that the two principal stresses in the horizontal direction are the maximum and minimum principal stresses, respectively, and the vertical stress is the intermediate principal stress. In addition, the vertical stress is approximately equal to the gravity stress. If we assume that the vertical stress is equal to the gravity stress, and the average unit weight γ is 26.50 kN/m^3^ for the rock mass, the vertical stress $\sigma_{V}=17.6\;\mathrm{MPa}$ can be calculated by $\sigma_{V}=H\gamma$. According to relevant research [42], *R*_1_ = 1.9 and *R*_2_ = 0.85, which are the ratio of horizontal maximum principal stress $\sigma_{\mathrm{Hmax}}$ and vertical stress, and minimum principal stress $\sigma_{\mathrm{Hmin}}$ and vertical stress. respectively, are determined in this study. Thus, the $\sigma_{\mathrm{Hmax}}=\sigma_{v}\times R_{1}=33.4\;\mathrm{MPa}$, $\sigma_{\mathrm{Hmin}}=\sigma_{v}\times R_{2}=14.8\;\mathrm{MPa}$ can be obtained.

**Figure 7:**
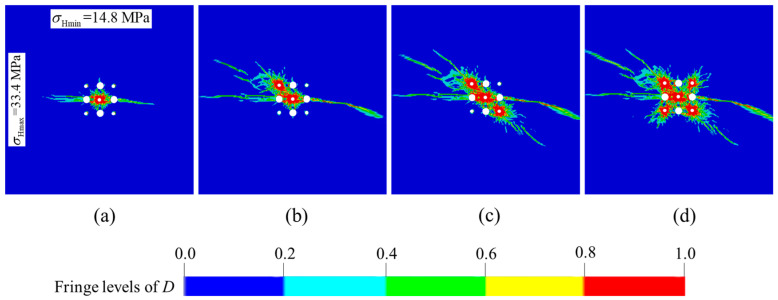
Damage evolution processes of burn cuts with the actual in-situ stress in Xincheng gold mine: (**a**) rock damage after the prime cut hole detonation; (**b**) rock damage after the No. 6 secondary cut holes detonation; (**c**) rock damage after the No. 8 secondary cut holes detonation; (**d**) rock damage after the No. 7 and 9 secondary cut holes detonation.

The numerical model in which the blasting parameters, the boundary conditions and the model size are consistent with Section 4.2 is built in ANSYS-LSDYNA. Only in-situ stress loading changes to the real values of the experimental stope in implicit analysis. In addition, the blasting processes of the four secondary cut holes are added to investigate the damage evolution mechanism of the overall cut cavity. The numerical results are shown in Figure 7. As we can see, a complete cut cavity is formed by a series of cut holes denotation in sequence. However, it can be found from Figure 7a that the damage zones between the prime cut hole and the empty holes are not interconnected, and that the burden rock did not break completely in the direction of the minimum principal stress. In practical engineering, this may lead to a failure of formation of the prime cut cavity, as it cannot provide the free surface and swelling space for the subsequent secondary cut holes. Therefore, the hole spacing needs to be optimized.

**Figure 8:**
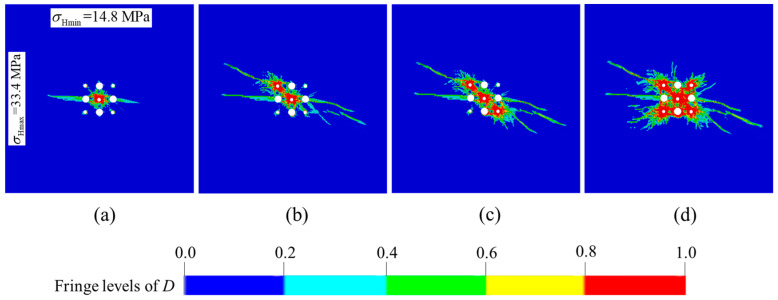
Damage evolution processes of the optimized burn cuts design by reducing hole spacing: (**a**) rock damage after the prime cut hole detonation; (**b**) rock damage after the No. 6 secondary cut holes detonation; (**c**) rock damage after the No. 8 secondary cut holes detonation; (**d**) rock damage after the No. 7 and 9 secondary cut holes detonation.

The hole spacings *L*_max_ are kept a constant with 250 mm in the direction of the maximum principal stress, while, the hole spacings *L*_min_ are reduced to 240 mm in the direction of the minimum principal stress. Meanwhile, the distance components of the corresponding secondary cut holes in the direction of the minimum principal stress are also reduced to 240 mm. The simulation results of cut model optimization are shown in Figure 8. It can be seen from Figure 8a that the damage zones are interconnected and that the cavity is in a “lotus” shape after the prime cut hole denotation. From the comparison of the final damage clouds between Figure 7d and Figure 8d, the area of *D* = 1 is larger, and the radial tensile cracks which extend to outside of the cut cavity are shorter in the optimization scheme than that in the original scheme. That means more blasting energy is spent in the creation of the cut cavity, and that the size of the rock fragments is smaller and they more easily fall out of the cut cavity in the optimization scheme. According to the above analysis, the hole spacings *L*_max_ = 250 mm and *L*_min_ = 240 mm for one-step raise excavation in the −665 m sublevel of Xincheng gold mine are determined.

## 5. Field Test

**Figure 9:**
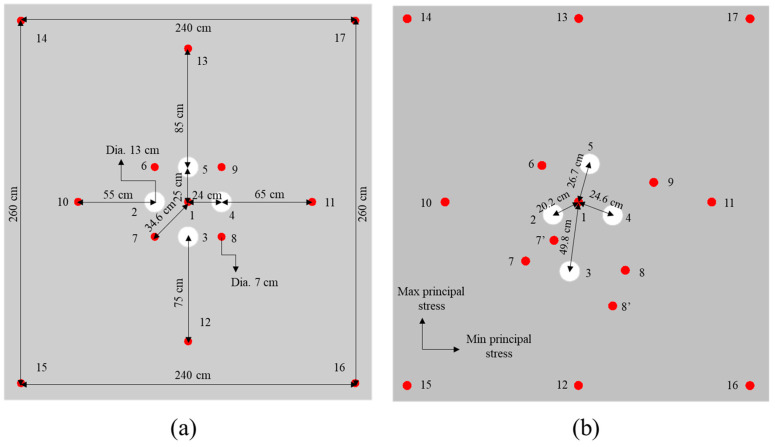
Borehole layout of burn cuts for the blind cut raise: (**a**) design scheme; (**b**) drilling result.

According to the drilling equipment selection and numerical simulation results, the cut parameters—such as hole diameters *d* = 70 mm, *Φ* = 130 mm and hole spacing *L*_max_ = 250 mm, *L*_min_ = 240 mm in the burn cuts with four empty holes—are determined. The in-situ stress measurement results show that the distribution of the maximum principal stress in Xincheng gold mine is close to the east-west trend per Cai et al. [41], while the trend of the V# ore body is north-south trend and the stope is arranged vertically, so that the direction of the maximum principal stress is relatively consistent with the length direction of the stope. Therefore, the hole spacing *L*_max_ is aligned with the length direction of the stope, while *L*_min_ is aligned with the width direction of stope. Moreover, the raise section is changed into a rectangle with a size of 2.4 × 2.6 m^2^ according to the in-situ stress distribution, as shown in Figure 9a. The layout of boreholes is consistent with Figure 1, and the parameters of hole spacings and diameters of boreholes are marked in detail. In addition, the depth of the empty holes is increased to 9.5 m to reduce the constriction at the top of the blind cut raise, which only has one free surface at the bottom.

The Sandvik drill rig with a drilling rate of 20 m/h is used to drill the boreholes based on the design drawing of the blind cut raise. However, it is difficult to locate accurately the point of boreholes due to dimly light and drilling straight up. Thereby, a serious borehole deviation appears between drilling and design drawing, as shown in Figure 9b. It can be seen that the distance between No. 3 (empty hole) and No. 1 (prime cut hole) is 498 mm, which is much larger than the design hole spacing of 250 mm. The corresponding burden rock may only perform a plastic deformation and not break into fragments. No. 3 empty hole cannot serve as the free surface and swelling space when the prime cut hole is initiated. Fortunately, although the distances from No. 2, 4, 5 to the prime cut hole have a deviation from the design (202 mm, 246 mm and 267 mm, respectively), it is still under control (swelling coefficient *k* > 1.45). In addition, it is found that there are two extra boreholes next to No. 7 and 8 secondary cut holes, which are named as No. 7′ and 8′, respectively.

The deviation of boreholes increases the difficulty of cut blasting in the BCBM. In order to solve this problem, some adjustments should be conducted. First, the charge density of cut holes is increased through reducing the air deck length. For cut holes No. 1, 7, 7′, 6, 9, 8 and 8′, the charge structure is set as 0.6 m explosive with 0.05 m air deck. Besides this, the stemming length is 0.2 m. The charge of each cut hole is 24.5 kg. Then a continuous charge structure is implemented in the supplementary holes and peripheral holes. For supplementary holes No. 10, 11, 12 and 13, the stemming length is 0.7 m and the charge is 38.3 kg. For peripheral holes No. 14, 15, 16 and 17, the stemming length is 1.0 m and the charge is 36.9 kg. Meanwhile, the bottom of the three empty holes, No. 2, 4, and 5, are filled with 0.2 kg explosive and 0.05 m stemming, respectively, to accelerate the removal of rock fragments from the cavity. 

**Figure 10:**
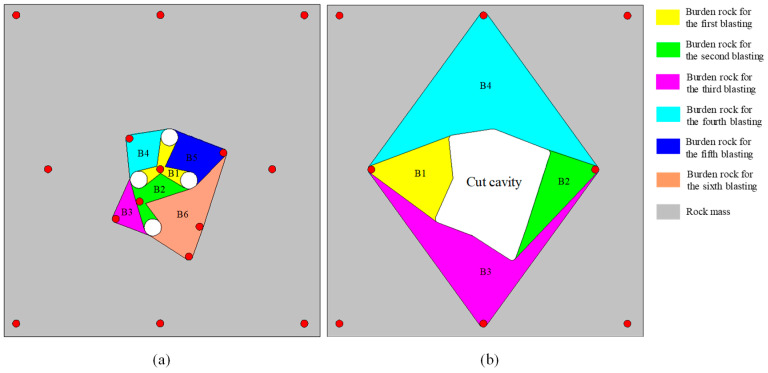
Adjustment for burn cuts with two steps: (**a**) detonation of cut holes in the first step; (**b**) detonation of supplementary and peripheral holes in the second step.

**Figure 11:**
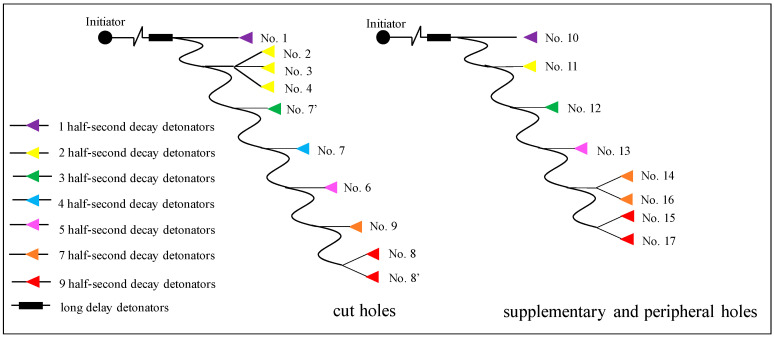
Initiation circuit of the 9 m blind cut raise with two steps.

After this, the BCBM is split into two steps: the cut holes are detonated first, after the cut cavity is formed, and then the supplementary holes and peripheral holes are detonated in sequence, as shown in Figure 10. According to the swelling space theory, the initiation sequence is determined as: No. 1→No. 2, 4 and 5→No. 7′→No. 7→No. 6→No. 9→No. 8 and 8′ at the first initiation, No. 10→No. 11→No. 12→No. 13→No. 14 and 16→No. 15 and 17 at the second initiation. The decay time of two adjacent boreholes t≥0.338 s is calculated; the detail calculation procedure refers to Liu et al. [4]. There are non-electric millisecond decay detonators and half-second decay detonators in the mine, and the decay time of the adjacent half-second detonator is 500 ms, so the half-second detonator is used to fire the blastholes, and the initiation circuit is determined based on delay times and initiation sequence, as shown in Figure 11. 

**Figure 12:**
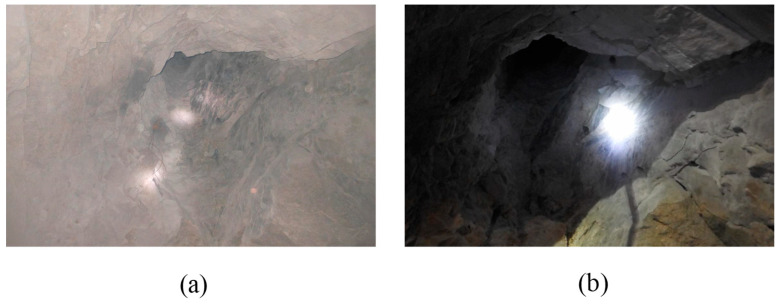
Blasting effect of the BCBM for the blind cut raise in deep: (**a**) cut cavity after the first initiation; (**b**) blind cut raise after the second initiation.

**Figure 13:**
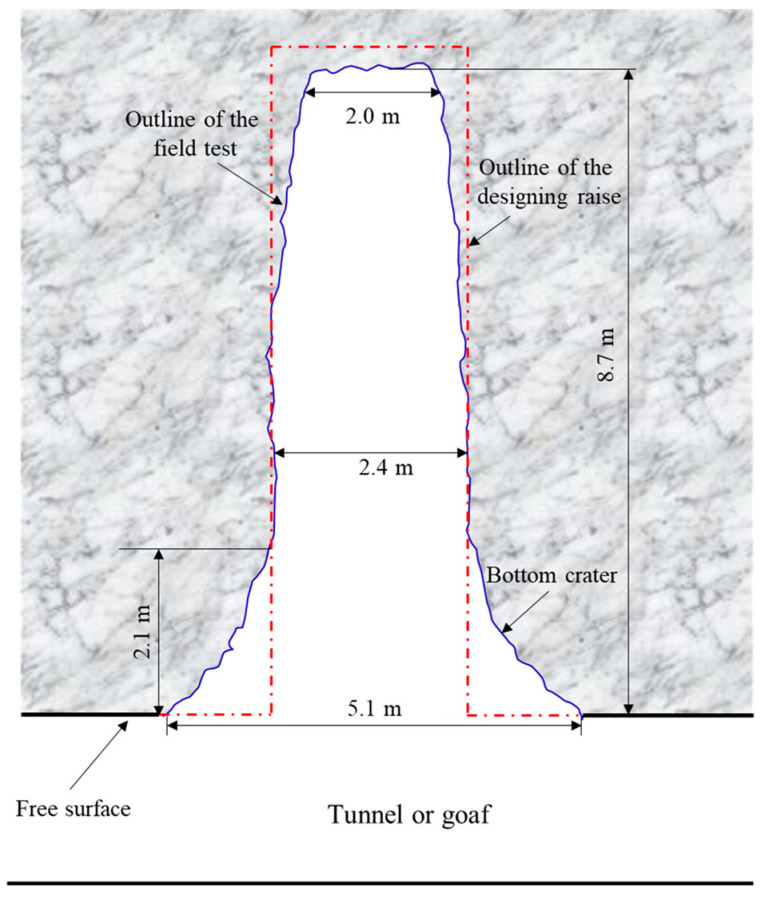
Profile of blind cut raise via cavity explorer scanned.

The blasting effect of BCBM for the blind cut raise in a deep mine is shown in Figure 12. As we can see, the result of the first detonation is presented in the Figure 12a, the cut cavity is formed completely with a crater at the mouth. Moreover, there is no blockage in the cavity, which can used as the free surface and swelling space for the subsequence boreholes. The Figure 12b shows the complete blind cut raise formed after the second detonation. It can be seen that the raise section is rectangular and the crater is expanded. Further, the cavity explorer of C-ALS is applied to scan the profile of the blind cut raise, as shown in Figure 13. The raise is smooth and free of clogging. The depth of 8.7 m basically reaches the design requirement. Meanwhile, the crater with 5.1 × 2.1 m^2^ (diameter × height) is formed at the bottom of the raise. The diameter of middle section of the raise is 2.4 m, which is consistent with the design. However, the diameter of the section at the top of the raise is only 2.0 m, less than the design value. The main reasons for this result is that there is a free surface at the bottom of the raise, which reduces the constriction of the burden rock, so a blasting crater is formed at the bottom of the raise. For the burden rock at the top of the raise, it is subjected to a high degree of constriction due to there being only empty holes as the free surface and swelling space. In order to overcome the high degree of constriction caused by a narrow free surface and high in-situ stress, explosive consumption can be increased at the top of the raise to form a more complete raise.

## 6. Discussion

The objective of this study is to investigate the damage evolution mechanism of deep cut blasting by using a tension-compression damage model. For cut blasting in deep mines, high in-situ stress has a very serious influence on rock damage evolution induced by blasting. Moreover, the rock constriction in cut blasting is higher than that in bench blasting [43]. Therefore, discovering how to solve the problem of cut blasting under high in-situ stress is urgent. Due to the efficiency, flexibility and low cost of computer algorithms, a numerical simulation method is used to study damage evolution mechanism of rock in this paper. Many damage models have been proposed to predict the damage evolution of rock in recent years [44,45]. However, most of the models, such as HJC or TCK, have a problem: the differences between tensile and compressive properties of rock materials cannot be described well. Thus, a damage model which considers the tensile and compressive damage separately is presented in this investigation. Meanwhile, two formulas are involved to describe strain rate effect under tension and compression, respectively. Moreover, the *J*_3_ is taken into account through introduction of the Lode-angle function.

The numerical results of cut blasting under different in-situ stresses show that the overall damage tends to decrease with the increase of in-situ stress, which is consistent with many researches. Separately, the tensile damage is reduced sharply while the compressive damage is increased under the action of in-situ stress. The trend of tensile and compressive damage can be intuitively seen by the new damage mode, which is conductive to analyze the effect of in-situ stress on tensile and compressive damage of rock. Further, the optimization cut parameters by numerical simulation of the new damage model are applied in a blind cut raise in deep mine. In the process of drilling, a deviation of boreholes between site operation and design is occurred. Therefore, a temporary adjustment was made to the design scheme: the one-step raise excavation is divided into two steps, and a better effect is obtained. In practical engineering, borehole deviation is a frequent phenomenon due to drilling operation or joints in rock mass. To ensure the success rate of one-step raise excavation, the field application should be flexible and changeable based on the actual situation.

In addition, the in-situ stress can enhance the compressive component of stress wave, which will result in the compressive damage increasing according to the above analysis. However, most of the papers are devoted to how to solve the problem of rock blasting suppression induced by in-situ stress in deep mines [7,14,26,46]. Some papers suggest using in-situ stress to expand the compressive damage and improve blasting effect. For large-scale rock blasting, the rock fragments are mainly produced by radial tensile cracks, so it is greatly affected by the negative effect of in-situ stress. As for the cut blasting in rock excavation, due to its small zone, the burden rock can be crushed by the shock wave induced by the explosion. Under the appropriate blasting parameters, the in-situ stress can promote the rock breaking in cut blasting. Therefore, it provides a direction for us to investigate how to use the promotion effect of in-situ stress on compressive damage in the future.

## 7. Conclusions

Due to the differences between tensile and compressive properties of rock material, an improved tensile-compressive damage blasting model based on the original HJC model is proposed to investigate cut blasting in deep raise. Then a numerical simulation of cut blasting under different in-situ stresses is carried out through the improved damage model, which is implemented into LS-DYNA software via user subroutines. The numerical results showed that the in-situ stress can promote the development of compressive damage while inhibiting the development of tensile damage. Subsequently, the cut parameters are optimized for blind cut raise in a specific project in Xincheng gold mine. Further, the optimization cut parameters are applied in the field test. The research conclusions are as follows:

1. An improved damage blasting model is proposed to describe the tensile and compressive damage separately, and then implemented into LS-DYNA to simulate the rock damage evolution in cut blasting for one-step raise excavation under different in-situ stresses.
2. In-situ stress causes resistance on the overall rock damage evolution induced by blasting stress wave. For compressive damage, the damage increases with the increase of in-situ stress, while for tensile damage, it decreases with the increasing in-situ stress. The underlying reason is that in-situ stress can superimpose the peak pressure of the compressive stress component, but weaken that of the tensile stress component.
3. The damage zones, including tensile and compressive damage, tend to develop along the direction of the maximum principal stress under the action of anisotropic in-situ stress. The phenomenon becomes distinctive as the difference between the two principal stresses increases, which is the main problem which should be solved in the deep cut blasting.
4. For a blind cut raise in −665 m of the Xincheng gold mine, the optimal cut parameters are obtained by numerical simulation results of the improved damage model. The blind cut raise with 8.7 m depth is successfully formed, which demonstrates that the optimization parameters are valid for one-step excavation in deep mines, and the adjustments of design are necessary for the actual situation.

## Figures and Tables

**Table 1:** Parameters of the improved damage model.

*ρ*/(kg/m^3^)	*G*/GPa	*K*/GPa	*B*	*N*	*f*_c_/MPa	*f*_t_/MPa	*D*_1_	*D*_2_
2810	17.2	33.2	2.57	0.62	133.5	6.76	0.04	1.0
***p*_c_/MPa**	***µ*_c_**	***p*_1_/GPa**	***µ*_1_**	***K*_1_/GPa**	***K*_2_/GPa**	***K*_3_/GPa**	***EF*_min_**	
44.5	0.0013	1.89	0.110	18.8	15.3	94.1	0.03	

**Table 2:** Parameters of explosive material and JWL EOS.

$\rho_{e}\;(\mathrm{kg}/m^{3})$	*VoD* (m/s)	*P*_CJ_ (GPa)	*A*_J_ (GPa)	*B*_J_ (GPa)	*R*_1_	*R*_2_	ω	*E*_0_ (GPa)
1210	5660	9.7	214.4	0.182	4.2	0.9	0.15	4.192

**Table 3:** In-situ stress loading stress for the cut blasting.

Loading Schemes	1	2	3	4	5	6	7	8
Horizontal stress in the x direction (MPa)	0	10	30	50	30	30	30	30
Horizontal stress in the z direction (MPa)	0	10	30	50	10	20	40	60

## Data Availability

No new data were created or analyzed in this study. Data sharing is not applicable to this article.
